# Impact of a Brief Educational Intervention on Parental Sun Safety Attitudes for Newborns

**DOI:** 10.1111/pde.70229

**Published:** 2026-04-27

**Authors:** Ayesha Kothari, Kaylee Gwyn, Bashir Khatib‐Shahidi, Faheed Shafau, Elizabeth Buzney

**Affiliations:** ^1^ Department of Dermatology Michigan State University College of Human Medicine Grand Rapids Michigan USA; ^2^ Department of Dermatology Brigham and Women's Hospital, Harvard Medical School Boston Massachusetts USA

**Keywords:** health education, newborn care, parental attitudes, skin cancer prevention, sun safety

## Abstract

Early‐life sun exposure accounts for a substantial portion of lifetime UV damage, with much of lifetime risk acquired before age 20 years. Despite clear pediatric and dermatology guidelines, many parents remain unsure of what optimal sun protection means, highlighting the need for accessible education and intervention. In our study, parents expressed strong interest in learning how to protect their newborns but lacked clear, actionable guidance. A brief, targeted educational session significantly improved parental attitudes toward sun safety and increased their intention to adopt recommended photoprotective behaviors.

## 1 Introduction

Skin cancer is the most common malignancy in the United States, and early‐life sun exposure significantly contributes to lifetime melanoma risk [1, 2, 3, 4]. Despite pediatric and dermatologic guidelines emphasizing strict sun protection during infancy [5, 6], there remain limited data on parental behaviors or counselling related to newborn sun safety. Infants are uniquely vulnerable due to immature melanocytes and a highly permeable epidermal barrier [7]. The American Academy of Pediatrics recommends sun avoidance and seeking shade as sun protection measures during infancy over sunscreen use, but these guidelines are not consistently communicated [5, 6].

## 2 Methods

We assessed whether a concise educational intervention could influence parental attitudes and intentions regarding sun protection for themselves and their newborns. An IRB‐approved, anonymous combined pre‐ and post‐survey study was conducted among parents attending a newborn care class. The session included a 20‐min presentation on UV risk, skin cancer prevention strategies, and infant‐specific protection recommendations, after which the survey assessing beliefs before and after the presentation was administered. Participants also received sun‐protective materials, including a UPF 50+ baby blanket.

## 3 Results

A total of 49 parents completed the pre‐intervention portion of the survey. Respondents were predominantly aged 26 to 35 (79.5%), with most (92.3%) identifying as first‐time parents. Over one‐third (35.9%) reported a personal or family history of skin cancer. Following the intervention, 43 participants completed both sections of the survey, while six participants did not finish the post‐intervention portion of the survey. Wilcoxon signed‐rank tests showed significant increases in skin cancer concern (*p* = 0.0022), likelihood of applying sunscreen to themselves (*p* < 0.0001), intention to protect their newborn from sun exposure (*p* = 0.0003), and intention to promote sunscreen use in their children (*p* = 0.0005). The proportion of respondents who reported being ‘somewhat’ or ‘very’ worried about developing skin cancer on a 5‐point Likert scale rose from 40% to 74%. Those likely to promote sunscreen use for their child increased from 56% to 90%, and intent to protect their newborn in the first 6 months rose from 62% to 82%. After the session 84% reported they were more likely to use sunscreen regularly, 100% planned to teach their child about sun safety, and 89.7% intended to practice sun protection with their newborn. Sun‐exposure history revealed that 66.7% had at least one blistering sunburn in childhood. Despite 79.5% reporting regular sunscreen use as children, 38.5% stated they never wear sunscreen in summer as adults, and 74.3% never wear it in winter. Forgetting to apply sunscreen was a universal barrier. Other deterrents included texture, cost, tanning preferences, and perceived lack of necessity. Motivators for sunscreen use included preventing sunburn (84.6%) and skin cancer (79.5%), with fewer citing aesthetic reasons such as aging (51.3%). Notably, only 7.7% of participants recalled ever receiving sun safety counseling for both themselves and their newborns, and 59% reported no prior counseling at all. Subgroup analysis revealed improvements across all demographic groups. Interestingly, non‐White participants showed significantly greater increases in intent to use sunscreen on their newborns than White participants (*β* = −1.00, *p* < 0.0001). No other demographic predictors—including gender, age group, or personal/family history of skin cancer—were significantly associated with changes in any outcome measure.

## 4 Discussion

Our findings suggest that short, structured educational sessions can significantly shift parental attitudes and increase their intentions to practice sun safety from the earliest stages of life. However, intention does not always lead to long‐term behavior change [8].

This study is limited by its small sample size, single‐institution setting and recruitment from a voluntary newborn injury prevention class, which may attract more health‐conscious or motivated parents than the general population. Additionally, while our subgroup analysis suggested greater improvements in sunscreen intentions among non‐White participants, the sample was not powered for robust comparisons across racial or ethnic groups. Future work should examine how demographic and cultural factors influence receptivity to sun safety education, as tailored approaches may be required to maximize impact across diverse populations.

Future studies should assess behavior sustainability with follow‐up at 6–12 months and explore integration into pediatric care. Simple strategies such as reminder systems, mobile apps, and culturally sensitive messaging could reinforce behavior [9]. Given time constraints in pediatric and prenatal visits, concise interventions with take‐home tools may help fill the current gap in counseling. Normalizing these conversations in routine newborn care could establish early habits that reduce lifetime skin cancer risk.

## Author Contributions

A.K. and K.G. conceived and designed the study. A.K. and K.G. acquired the data. B.K.‐S. and F.S. analysed the data. A.K. drafted the manuscript, and all authors critically revised it and approved the final version.

## Consent

All persons gave their informed consent prior to their inclusion in the study.

## Conflicts of Interest

The authors declare no conflicts of interest.

## Supporting information


**Data S1:** pde70229‐sup‐0001‐Supinfo.pdf.

## Data Availability

The data that support the findings of this study are available from the corresponding author upon reasonable request.
