# Supplementary material for: Impact of a Brief Educational Intervention on Parental Sun Safety Attitudes for Newborns
Source: Pediatr Dermatol. 2026 Apr 27;43(5):1152–3. doi: 10.1111/pde.70229 (PMC13617174; doi:10.1111/pde.70229)
Supplement: Supplementary file 1 [file PDE-43-1152-s001.pdf]

Consent to Participate

You indicate your voluntary agreement to participate by completing and submitting this survey. You must be 18 or older to continue taking this survey. This survey will take approximately **5 minutes** to complete.

○ Agree

Did you attend the Sun Safety for Newborns and New Parents education session with the Injury Prevention team at Helen DeVos?

○ Yes
○ No

What day did you attend the Sun Safety for Newborns and New Parents education session?

[1/7/2026]

Demographics

What gender describes you?

○ Male
○ Female
○ Non-binary
○ [ ] Other

How old are you?

○ 18-20
○ 21-25
○ 26-30
○ 31-35
○ 36-40

How would you describe your race?

○ White
○ Black or African American
○ Hispanic or Latino
○ Asian
○ American Indian or Alaska Native
○ Native Hawaiian or Other Pacific Islander
○ Middle Eastern or North African
○ Multiracial or Mixed Race
○ [ ] Other (please specify)

Is this your first child?

○ Yes
○ No

Do you have a personal or family history of skin cancer?

○ Yes
○ No

Do you know someone who has had skin cancer?

○ No
○ Yes (If so, who? i.e. neighbor, grandparent, sibling, etc)
[ ]

Has your primary care doctor or pediatrician ever discussed **sun safety** with you?

○ Yes, for myself
○ Yes, for my newborn/children
○ Yes, for both myself and my newborn/children
○ No
○ [ ] Other

Has your primary care doctor or pediatrician ever discussed **skin cancer prevention** with you?

○ Yes, for myself
○ Yes, for my newborn/children
○ Yes, for both myself and my newborn/children
○ No
○ [ ] Other

Personal Data on Sunscreen

Did you wear sunscreen as a child?

○ Yes, daily
○ Yes, only on sunny days
○ No

As a child, do you recall having at least 1 blistering sunburn?

○ Yes
○ No

In the summer, how often do you wear sunscreen?

○ Never
○ Once a month
○ Once a week
○ 2-3 times a week
○ Every day

In the winter, how often do you wear sunscreen?

○ Never
○ Once a month
○ Once a week
○ 2-3 times a week
○ Every day

How often do you reapply sunscreen?

○ Every 1-2 hours
○ Every 3-4 hours
○ Once a day
○ Never

What influences whether or not you wear sunscreen?

☐ UV index
☐ Visible sun outside
☐ Whether friends/family are wearing it
☐ [ ] Other

What discourages you from wearing sunscreen?

☐ Wanting a tan
☐ Oily/greasy feeling
☐ Forget to apply
☐ Don't think you need it
☐ Cost
☐ Friends/family aren't wearing it
☐ [ ] Other

How do you prefer to protect yourself from the sun?

☐ Sunscreen
☐ Clothing (including hat, sunglasses, etc.)
☐ Staying out of the sun
☐ [ ] Other

What motivates you to protect yourself from the sun?

☐ Slowed aging of skin (wrinkles, sun spots)
☐ Preventing skin cancer
☐ Preventing sunburns
☐ [ ] Other

What type of sun protective behaviors do you exercise? (select all that apply)

☐ Avoid sun exposure during peak hours (10 am-2 pm)
☐ Wear hats
☐ Seek shade/use an umbrella
☐ Wear sunglasses
☐ Wear UPF clothing
☐ Minimize exposed skin
☐ Annual skin exams with your dermatologist
☐ Monthly self skin exams
☐ [ ] Other

On average, how many sunburns do you get in a year?

○ 0
○ 1
○ 2
○ 3
○ 4
○ 5+

Where did your previous knowledge of sun safety come from?

○ I have no knowledge on sun safety
○ Dermatologist/Primary Care Physician
○ Parent
○ Friend
○ Significant Other
○ Social media
○ Peer-reviewed literature
○ [ ] Other

Before & After Sun Safety Education

Prior to this presentation, how worried **were you** about developing skin cancer?

○ Not worried at all
○ Aware of risk but not worried
○ Neutral
○ Somewhat worried
○ Very worried

After this presentation, how worried **are you** about developing skin cancer?

○ Not worried at all
○ Aware of risk but not worried
○ Neutral
○ Somewhat worried
○ Very worried

Prior to this presentation, how likely **were you** to promote sunscreen use in your children?

○ Extremely unlikely
○ Somewhat unlikely
○ Neither likely nor unlikely
○ Somewhat likely
○ Extremely likely

After this presentation, how likely **are you** to promote sunscreen use in your children?

○ Extremely unlikely
○ Somewhat unlikely
○ Neither likely nor unlikely
○ Somewhat likely
○ Extremely likely

Prior to this presentation, how likely **were** you to protect your **newborn** from the sun during their first 6 months of life?

○ Extremely unlikely
○ Somewhat unlikely
○ Neither likely nor unlikely
○ Somewhat likely
○ Extremely likely

After this presentation, how likely **are** you to protect your **newborn** from the sun during their first 6 months of life?

○ Extremely unlikely
○ Somewhat unlikely
○ Neither likely nor unlikely
○ Somewhat likely
○ Extremely likely

Prior to this presentation, how likely **were** you to protect **yourself** from the sun throughout the next 6 months?

○ Extremely unlikely
○ Somewhat unlikely
○ Neither likely nor unlikely
○ Somewhat likely
○ Extremely likely

After this presentation, how likely **are** you to protect **yourself** from the sun throughout the next 6 months?

○ Extremely unlikely
○ Somewhat unlikely
○ Neither likely nor unlikely
○ Somewhat likely
○ Extremely likely

Overall, this presentation made me ... (select all that apply)

☐ More aware of skin cancer risks
☐ Plan to use sunscreen more often
☐ Plan to teach my kids about sun safety and skin cancer risks
☐ Plan to practice sun safety with my newborn
☐ [ ] Other

Quality Improvement

If you would be willing to fill out a follow-up survey in 6 months, please include your email below. We strongly appreciate your support.

[${m://Email1}]

Have you ever visited the Injury Prevention website?

○ Yes
○ No

How often do you anticipate you will use the Coolibar Baby Sun Blanket provided?

○ Every single day
○ Most days
○ Some days
○ Only on sunny days
○ Never

How can we improve this presentation and experience?

[ ]

Is there anything else you'd like us to know?

[ ]

Questions? Comments? Concerns?

[ ]

Powered by Qualtrics
